# Paraneoplastic Syndromes in Hepatocellular Carcinoma, Epidemiology, and Survival: A Retrospective Seven Years Study

**DOI:** 10.3390/medicina60040552

**Published:** 2024-03-28

**Authors:** Calin Burciu, Roxana Sirli, Renata Bende, Deiana Vuletici, Bogdan Miutescu, Tudor Moga, Felix Bende, Alina Popescu, Ioan Sporea, Oana Koppandi, Eftimie Miutescu, Dana Iovanescu, Mirela Danila

**Affiliations:** 1 Department of Gastroenterology and Hepatology, “Victor Babes” University of Medicine and Pharmacy Timisoara, Eftimie Murgu Square 2, 300041 Timisoara, Romania; calin.burciu@umft.ro (C.B.); renata.fofiu@yahoo.com (R.B.); deiana.vuletici@umft.ro (D.V.); miutescu.bogdan@umft.ro (B.M.); moga.tudor@gmail.com (T.M.); bendefelix@gmail.com (F.B.); popescu.alina@umft.ro (A.P.); isporea@umft.ro (I.S.); mireladanila@gmail.com (M.D.); 2 Advanced Regional Research Center in Gastroenterology and Hepatology, “Victor Babes” University of Medicine and Pharmacy Timisoara, 300041 Timisoara, Romania; 3 Department of Gastroenterology, Faculty of Medicine, Pharmacy and Dental Medicine, “Vasile Goldis” West University of Arad, 310414 Arad, Romania; oanakoppandi@yahoo.com (O.K.); emiutescu@yahoo.com (E.M.); danagastro@yahoo.com (D.I.)

**Keywords:** hepatocellular carcinoma, paraneoplastic syndromes, survival

## Abstract

*Background and Objectives:* Liver cancer poses a significant global health threat, ranking among the top three causes of cancer-related deaths. Patients with hepatocellular carcinoma (HCC) often present with symptoms associated with neoplasms or unusual clinical features such as paraneoplastic syndromes (PNS), including hypoglycemia, hypercholesterolemia, thrombocytosis, and erythrocytosis. Our study aimed to investigate the prevalence, clinical characteristics, and survival outcomes associated with PNS in HCC patients and assess each PNS’s impact on patient survival. *Materials and Methods:* We conducted a retrospective analysis of PNS clinical features and survival among consecutive HCC patients diagnosed at our department over seven years, comparing them with HCC patients without PNS. The study involved a retrospective data evaluation from 378 patients diagnosed with HCC between January 2016 and October 2023. *Results:* We obtained a PNS prevalence of 25.7%, with paraneoplastic hypercholesterolemia at 10.9%, hypoglycemia at 6.9%, erythrocytosis at 4.5%, and thrombocytosis at 3.4%. Patients with PNS tended to be younger and predominantly male. Multivariate analysis revealed a strong correlation between PNS and levels of alpha-fetoprotein and tumor size, with diabetes also showing a significant statistical association (*p* < 0.05). Subgroup analysis based on specific paraneoplastic syndromes demonstrated shorter survival in patients with PNS, albeit without significant statistical differences, except for hypoglycemia (*p* < 0.0001). Matched analysis indicated a shorter survival rate for patients with PNS, although no significant statistical differences were observed. *Conclusions:* PNS are frequently observed in HCC cases and are associated with unfavorable prognoses and decreased survival rates due to their correlation with increased tumor burdens. However, they do not independently predict poor survival. The impact of individual PNS on HCC prognosis varies.

## 1. Introduction

Liver cancer is a pressing global health concern as it affected an estimated 905,700 individuals in 2020, leading to 830,200 fatalities worldwide. HCC ranks among the top three causes of cancer-related fatality in 46 countries and within the top five in 90 countries. Disturbingly, projections indicate a worrying trend for the future: between 2020 and 2040, the incidence of liver cancer is anticipated to surge by a staggering 55.0% [1]. Hepatocellular carcinoma (HCC) is thought to account for 80% of all liver cancers detected worldwide [2].

Roughly 90% of HCCs are linked to underlying risk factors. The most common ones include chronic viral hepatitis (types B and C), alcohol consumption, metabolic-associated fatty liver disease (MAFLD), and exposure to aflatoxins [3]. Cirrhosis is a significant risk factor for HCC, with approximately one-third of individuals developing HCC during their lives [4]. The prognosis for HCC patients hinges on early tumor detection, which relies on effective surveillance programs.

Patients with HCC often exhibit various symptoms and typically present with structural discomforts like weakness, abdominal mass, and weight loss. Additionally, patients may display atypical clinical features related to paraneoplastic symptoms [5]. Moreover, they may show signs of liver decompensation and right abdominal discomfort due to an enlarging liver mass. Hypercholesterolemia, hypoglycemia, hypercalcemia, and erythrocytosis stand out as the predominant paraneoplastic syndrome (PNS) detected in cases of HCC [6,7,8]. While PNS encompassing rheumatic, neuromuscular, dermatological, hematological, and endocrine disorders are less frequent [9,10], they share a common origin.

These paraneoplastic syndromes typically arise from the body’s reaction to the tumor’s abnormal metabolism or the release of the bioactive molecules produced by the tumor [11]. PNS manifests in approximately 16.8% to 43.6% of individuals diagnosed with HCC [5,12,13,14] and has been correlated with accelerated tumor advancement, the development of sizable tumors, the occurrence of multiple tumors within the liver, elevated serum levels of alpha-fetoprotein (AFP), and the invasion of HCC into the portal vein [8]. PNS among HCC patients have been linked to increased morbidity and poorer survival outcomes [13]. Nevertheless, specific reports have suggested that erythrocytosis and thrombocytosis may be associated with a more favorable prognosis [14].

Another paraneoplastic syndrome observed in HCC patients includes cutaneous manifestations, though they are less common. Dermatomyositis, pityriasis rotunda, and porphyria are the most frequently reported skin lesions. These manifestations are often more prevalent in patients who have a more significant disease burden, especially those with metastatic involvement. Notably, a substantial improvement in these skin conditions is typically seen after receiving treatment aimed at the HCC [15].

This study has two primary objectives: first, to investigate the prevalence of PNS in Romanian patients diagnosed with HCC, and second, to assess the influence of these syndromes on patient survival.

## 2. Materials and Methods

### 2.1. Study Populations

This retrospective study examined data from 378 patients who received a diagnosis of HCC between January 2016 and October 2023 at the Gastroenterology Clinic of Timisoara County Hospital. Information encompassing clinical, laboratory, and imaging data was retrieved from the hospital’s data system, which included variables such as age, gender, the complete blood count, liver biochemistry, AFP levels, Child–Pugh scores, the model for end-stage liver disease (MELD) scores, tumor volume, portal vein thrombosis, and the presence of diabetes mellitus. We also assessed subsequent hospitalizations for the appearance of PNS. Patients were followed up on from the moment they were diagnosed until 1 October 2023, when a survival census was conducted.

The study received approval from the ethics committee of Timișoara County Hospital under the reference number 234/28 September 2023, and it adheres to the principles outlined in the Declaration of Helsinki.

### 2.2. Diagnosis of Hepatocellular Carcinoma

The diagnosis of HCC was established following the guidelines set forth by the European Association for the Study of the Liver (EASL) [16], which requires the presence of a focal liver lesion displaying hypervascularity during the arterial phase and subsequent washout during the portal venous and/or delayed phases, as determined by contrast-enhanced ultrasound (CEUS), contrast-enhanced computed tomography (CE-CT), or contrast-enhanced magnetic resonance imaging (CE-MRI) [17]. A biopsy of the tumor was performed for the lesions with atypically imagistic aspects and with a high suspicion of HCC. The staging of HCC was carried out using the Barcelona Clinic Liver Cancer (BCLC) classification method [18]. Liver cirrhosis was diagnosed through a combination of clinical observations, liver elastography (utilizing FibroScan^®^, Echosens^TM^, Paris, France), and/or biomarker assessments (FibroTest-ActiTest, FibroMax).

### 2.3. Laboratory Testing

AFP levels were assessed by analyzing venous blood; 5 mL of blood was collected from fasting patients using a vacutainer devoid of anticoagulants. After allowing the blood to clot at room temperature, the serum was separated using centrifugation within the first four hours post-collection. At least 0.5 mL of serum was required for the analysis. The concentration of AFP in the serum was then measured using either a VITROS^®^ XT 7600 or a VITROS^®^ XT 3600 (Illkirch, France) device, which can detect AFP concentrations as quickly as 0.24 ng/mL.

Venous blood was collected and processed similarly to the AFP detection sample to determine cholesterol and glucose levels. Quantification was performed using the VITROS^®^ XT 7600 system. Erythrocytes and platelet levels were assessed by collecting venous blood into EDTA tubes and subsequently analyzing it with the 9100 Nihon Kohden Celltac (Tokyo, Japan) device.

### 2.4. Definition of Paraneoplastic Syndromes

Without established diagnostic criteria for PNS in HCC, we adopted the following criteria based on the existing literature. Erythrocytosis was defined as a hemoglobin level exceeding 16.5 g/mL or an RBC count exceeding 5.5 × 10^12^/L for males, and for females, it was indicated by a hemoglobin level surpassing 15 g/mL or an RBC count greater than 5.0 × 10^12^/L. Importantly, we systematically ruled out conditions such as polycythemia vera and myeloproliferative diseases in both genders [8]. Hypoglycemia was characterized by a plasma glucose level below 64.98 mg/dL, hypercholesterolemia was established when the serum cholesterol level exceeded 220.42 mg/dL, and thrombocytosis was identified by a platelet count exceeding 400 × 10^9^/L [14].

It is worth noting that while hypercalcemia is a common occurrence in HCC patients with paraneoplastic syndromes [14], we could not include these data in our study due to the limited number of patients who underwent the necessary laboratory analyses.

### 2.5. Statistical Analysis

Data analysis was conducted using MedCalc Version 19.4 (MedCalc Software Corp., Brunswick, ME, USA) and Microsoft Office Excel 2019 (Microsoft for Windows). Descriptive statistical methods were applied to the patients’ clinical data. The distribution of numerical variables was determined using the Kolmogorov–Smirnov test. Continuous variables with a normal distribution were presented as mean and standard deviations (SD), while those with a non-normal distribution were presented as the median and interquartile range (IQR).

Categorical variables were expressed as counts (percentages). To compare groups, the Kruskal–Wallis H test was utilized, followed by post-hoc analysis with the Mann–Whitney U test applying Bonferroni correction. The Student’s *t*-test was used to compare continuous variables with a normal distribution, while the Mann–Whitney U-test was applied for variables with a non-normal distribution.

Uni-and multivariate regression analyses were conducted to evaluate the independent predicting factors associated with the presence of PNS. The Akaike information criteria were used to determine the best regression model. Kaplan–Meier survival was used to estimate the probability of survival over time and for the calculation of median survival times. A *p*-value of less than 0.05 was considered statistically significant.

## 3. Results

### 3.1. Frequency of Paraneoplastic Syndromes in Individuals with Hepatocellular Carcinoma

The present study was conducted on 378 patients, with ages ranging from 32 to 93 years old, and a mean age of 64.86 ± 8.85 years, including 70.6% men (257) and 29.4% women (111), all of whom were diagnosed with HCC. The cohort was divided into two groups according to the presence of paraneoplastic syndromes, as follows: the PNS-positive group (*n* = 97) and PNS-negative group (*n* = 281). The PNS-positive subgroup represented 25.7% of the total HCC patients (hypoglycemia—6.9%, erythrocytosis—4.5%, hypercholesterolemia—10.9%, and thrombocytosis—3.4%). Notably, the PNS-positive patients, with an average age of 64.2 ± 8.35 years, exhibited a higher proportion of males (82.5%) compared to the overall distribution of males among HCC patients (70.6%) (*p* = 0.0226).

Examining the distribution of subjects based on the Child–Pugh score revealed a higher prevalence of the Child–Pugh C stage in the PNS-positive group (31.8%), indicating an association between PNS positivity and a poorer prognosis in HCC. The analysis also demonstrated that 90.7% of PNS-positive HCC cases developed on a cirrhotic background, while vascular invasion occurred in 41.2% of PNS-positive patients, in contrast to 30.6% in PNS-negative patients (*p* = 0.0731).

The baseline characteristics, demographic data, and laboratory parameters of the included subjects are summarized in Table 1 and Table 2.

The PNS-positive group exhibited significantly higher alpha-fetoprotein levels, approximately five times, compared to the PNS-negative group (*p* < 0.0001).

The associations between certain laboratory parameters and prognosis were identified. Hypoglycemia (27.3%) correlated with a higher rate of poor prognosis in the Child C group, while erythrocytosis (19.3%) and hypercholesterolemia (43.2%) were associated with a better prognosis in the Child A–B group. The distribution of subjects according to the Child–Pugh stage is summarized in Table 3.

Within the PNS patients, hypoglycemia (26.8%), erythrocytosis (17.5%), hypercholesterolemia (42.3%), and thrombocytosis (13.4%) were identified. Etiologically, PNS-positive cases were more prevalent in C virus hepatitis etiology (33%) compared to the lowest rate in MASLD etiology (13.4%). The distribution of subjects according to etiology is summarized in Table 4.

Multivariate analysis highlighted the significance of parameters such as albumin, total bilirubin, AFP, the presence of diabetes mellitus, and a tumor size < 5 cm in the PNS-positive group compared to the PNS-negative group. The extended results are presented in Table 5. A more detailed analysis was performed according to the type of paraneoplastic syndromes and is summarized in Table 6.

### 3.2. Patients’ Survival

**Figure 1:**
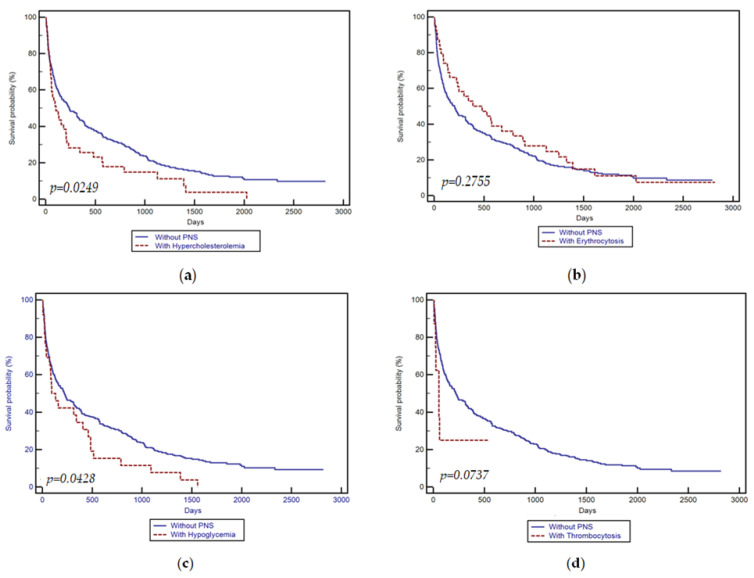
Kaplan–Meier survival curve according to the presence of paraneoplastic syndromes: (**a**) hypercholesterolemia; (**b**) erythrocytosis; (**c**) hypoglycemia; (**d**) thrombocytosis.

Among patients with paraneoplastic syndromes, the median survival time was 145 (1–2819) days, compared with 208 (1–2819) days, in subjects without paraneoplastic syndromes (*p* = 0.3286). Kaplan–Meier’s survival analyses are summarized in Table 7, while the survival curve is illustrated in Figure 1a–d.

Ninety-seven matched subjects from the group of subjects without paraneoplastic syndromes were selected. Subjects were matched based on their age, sex, biological test, Child–Pugh grade, and BCLC classification.

A subgroup analysis based on a paraneoplastic syndrome revealed significant differences in patients with erythrocytosis compared to those without paraneoplastic syndromes in terms of tumor size, albumin, and AFP levels (*p* < 0.001). However, despite these differences, no significant differences were found between the median survival time in patients with erythrocytosis, compared to those without paraneoplastic syndromes (480 vs. 204 days, *p* = 0.2755; Figure 1). In a matched analysis of 97 pairs with similar baseline characteristics, this aspect remained the same.

Patients with hypoglycemia differed significantly from those without paraneoplastic syndromes in terms of age and tumor size (*p* < 0.0001). Despite being younger and with a larger tumor size, those with hypoglycemia showed significantly shorter median survival compared to patients without paraneoplastic syndromes (130 vs. 220 days, *p* = 0.0428; Figure 1). However, in the matched analysis, no significant differences were found.

The analysis of patients with thrombocytosis before matching did not reveal a significant difference in the median survival time compared to patients without any paraneoplastic syndromes (53 vs. 220 days, *p* = 0.0737; Figure 1). Similar results were obtained in a comparison of patients with thrombocytosis to matched patients without paraneoplastic syndromes (61 vs. 233 days, *p* = 0.0831).

## 4. Discussion

HCC presents a significant global health challenge, and Romania is no exception, ranking second in Europe in terms of both incidence and mortality [18]. Thus, our study was initiated to offer further insights into the characterization of HCC, mainly focusing on PNS and their impact on survival.

In our cohort, the incidence of PNS was 25.7%, closely resembling the findings of the most recent study by Ülger et al., which reported 21% [14]. Moreover, our study aligns well with previous research predominantly originating from Asia, where the prevalence of PNS ranges between 27.8% and 30.9% [6,13]. Feng et al. reported the lowest incidence at 18.7%, although their study only included patients who underwent hepatic resection [8]. Therefore, considering that PNS is correlated with advanced liver disease and the progression of HCC, patients who benefit from liver resection typically present with early-stage HCC and preserved liver function.

The most common PNS in our cohort was hypercholesterolemia (10.9%), mirroring the findings of studies conducted by Chang in 2013 and Qu in 2014, albeit with substantially higher percentages of 24.5% [6] and 23.2% [13], respectively. Interestingly, in the study by Ülger et al., hypercholesterolemia emerged as the rarest PNS, accounting for only 2.4% [14]. Hypoglycemia followed as the second most prevalent PNS at 6.9%, trailed by erythrocytosis at 4.5%, with thrombocytosis being the least common at 3.4%.

The results of our study unveiled a correlation between the presence of PNS and older age, with a predominance of male patients at 82.5% (*p* = 0.0226), compared to the overall male distribution among HCC patients, which stands at 70.6%. However, it is crucial to interpret this high proportion of males in the context of the established male-to-female incidence ratio of HCC, typically ranging from 2 to 2.5 to 1 [19]; in our study, it is 2 to 1. Additionally, we observed a higher prevalence of the Child–Pugh C stage among the PNS-positive group (31.8%). Concurrently, vascular invasion was identified in 41.2% of PNS-positive patients, as opposed to 30.6% in PNS-negative patients. Furthermore, the PNS-positive group exhibited significantly elevated levels of AFP compared to the PNS-negative group (*p* < 0.0001). These factors, including manifestation in the advanced stage of liver diseases, heightened AFP values, and the association with vascular invasion, collectively contribute to poor survival outcomes in patients with PNS, confirming the conclusions drawn by Chang et al. in their study [6]. Markedly elevated levels of AFP indicate heightened biological activity within the malignant hepatocytes, which can produce various proteins, leading to PNS. Nevertheless, evidence suggests that treating HCC can alleviate PNS [20]. Paraneoplastic activity correlates with the extent of tumor burden and the biological vigor of the malignant hepatocytes, potentially resurfacing alongside HCC recurrence [21].

Regarding the etiology of the underlying disease, we observed that PNS has a higher incidence in patients with HBV infection (27.8% vs. 17.1%, *p* < 0.05), as confirmed in previous studies [5,14], but contradicts Chang’s study, where no significant differences were observed between patients with PNS and those without [6]. In the multivariate analysis, we noted that PNS was strongly associated with the AFP value and an HCC size of over 2 and 5 cm and with the BCLC class, indicating a correlation with tumor activity. Interestingly, diabetes mellitus was associated with PNS, albeit with statistical significance only, without a strong correlation (*p* < 0.026).

In our cohort, we observed a higher prevalence of patients with hypercholesterolemia in the group with paraneoplastic syndromes and HBV infection compared to those with HCV, at 51.8% versus 37.5%, as confirmed by Ülger et al. [14]. Additionally, approximately half of the patients were classified as Child–Pugh Class A, which weakens the argument that advanced liver disease is strongly linked to the emergence of PNS. The development of hypercholesterolemia in patients with HCC is likely attributed to the disruptions in cholesterol synthesis within tumor cells, characterized by the loss of regulatory mechanisms and the increased activity of the enzymes involved in cholesterol production [13]. Furthermore, aside from disrupting negative feedback mechanisms, HCC cells can independently boost de novo cholesterol synthesis by upregulating the expression of HMG-coA reductase within tumor cells [22]. Additionally, a genetic predisposition associated with a metabolic syndrome may influence the occurrence of hypercholesterolemia in HCC patients; further studies are needed to confirm this hypothesis. This hypothesis gains support from the observation in our study that, although not strongly correlated, diabetes showed statistical significance with PNS. Furthermore, diabetes remained statistically significant in hypercholesterolemia’s multivariate analysis (*p* < 0.026). Moreover, a correlation was noted between hypercholesterolemia and the AFP value, notably strongly associated with a tumor size exceeding 5 cm, indicating that hypercholesterolemia is more closely associated with tumor size.

Hypoglycemia, the second most frequent PNS in our cohort (6.9%), with the most recent data indicating an incidence between 5.8% and 13.1% [8,13,14], is more prevalent in China than Europe. Most often, it is discovered because of clinical symptoms appearing in the terminal phases of the disease when the tumor burden is high. The appearance of hypoglycemia involves two mechanisms [13]. In cases of type A hypoglycemia, the underlying mechanism involves the inability of the liver, tumor, and other tissues to adequately supply glucose stemming from liver failure, reduced hepatic glucose output due to malnutrition, and an elevated glucose demand by the tumor [14]. On the other hand, type B hypoglycemia in HCC manifests earlier in the disease progression and constitutes only a tiny percentage of paraneoplastic hypoglycemia cases in HCC [23]. Tumor tissue from type B hypoglycemic patients have exhibited markedly higher insulin-like growth factor (IGF) II mRNA levels—up to 10–20 times greater than those found in normal liver tissue [24]. Supporting the notion that hypoglycemia is associated with the terminal stage of liver disease is our study in which 50% of PNS in Child–Pugh C patients were hypoglycemia. In the multivariate analysis, hypoglycemia was strongly associated with high AFP values (*p* < 0.0001) and also with diabetes, even if the latter only had statistical significance (*p* < 0.038).

Erythrocytosis emerged as the third most frequent PNS in our study, with a prevalence of 4.5%. Other studies have reported a prevalence ranging from 3.9% to 10.3% [6,8,13,14]. The production of erythropoietin by the tumor itself has been identified as the most likely mechanism [25]. Additionally, erythropoiesis may act as a compensatory response to extensive tumor growth, overwhelming vascular support and resulting in local ischemia and hypoxia [26]. Our study observed erythrocytosis in 30.8% of cases with PNS in Child–Pugh Class A, notably decreasing as the disease progressed: 19.1% in Child–Pugh B and 3.6% in Child–Pugh C, respectively. These findings closely align with those of previous studies [8,14], suggesting that erythrocytosis may not negatively impact prognosis [13] and could potentially even enhance it [8].

Thrombocytosis was the least frequent PNS in our study (3.4%), with an incidence similar to other studies ranging from 1.7% to 3.9% [8,14]. Thrombocytosis occurring in HCC is an uncommon paraneoplastic phenomenon linked to the release of thrombopoietin (TPO) or the megakaryocyte growth factor from both hepatocytes and bone marrow cells [27]. There is a suggestion that thrombocytosis associated with HCC stems from the production of TPO by the tumor cells [28]. In our study, the distribution among the etiologies and the Child–Pugh Class was relatively similar; moreover, in the multivariate analysis, no parameter was identified to have a close correlation. Thus, it is likely that the occurrence of thrombocytosis is influenced by factors other than those we studied, which could be a potential avenue for future research.

Most studies have observed that PNS is associated with a shorter survival time. Qu et al. reported a median survival rate of 15 months for patients with PNS compared to 55 months for those without PNS (*p* = 0.003) [13]. In another report, Chang et al. found a difference in the median survival of 12.4 weeks versus 18.3 weeks in patients without PNS (*p* = 0.025) [9]. Similar data were also found in our study, indicating that the median survival time was 145 days for patients with PNS compared to 208 days for those without PNS (*p* = 0.3286). However, previous reports did not utilize propensity score matching.

We observed statistical differences in age, gender, serum AFP level, etiology, and the Child–Pugh score between patients with and without PNS and decided to conduct propensity score matching. We conducted a subgroup analysis focusing on PNS, which unveiled notable distinctions between patients exhibiting erythrocytosis and those without such syndromes regarding tumor size, albumin levels, and AFP levels (*p* < 0.001). Despite a shorter survival time, no statistically significant variance was observed in the median survival time between patients with erythrocytosis and those without it (*p* = 0.2755). This finding persisted even after conducting a matched analysis with comparable baseline characteristics. These results align with the data published by Qu et al. and Chang et al. [6,13], who did not find a significant reduction in the average survival time in patients with erythrocytosis. The only study reporting better survival rates in patients with erythrocytosis was published by Feng et al., focusing on patients who underwent liver resection. Thus, erythrocytosis serves as a favorable prognostic factor associated with survival only in patients undergoing therapeutic techniques with curative intent, such as liver resection. An explanation for this could be a lower pre-operative blood transfusion requirement in patients with erythrocytosis, suggesting that these patients were more tolerant of intra-operative blood loss and thus had better post-resection survival rates [8]. Similarly, their findings were derived from HCC tumors deemed suitable for resection. This might limit their generalizability to the broader population of HCC tumors accompanied by PNS, which are frequently more advanced and unsuitable for surgical resection.

Patients with hypoglycemia tend to be younger and have larger tumor sizes; those with hypoglycemia showed significantly shorter median survival compared to patients without PNS (*p* = 0.0428). However, in the matched analysis, no significant differences were found. The survival rate in patients with hypercholesterolemia and thrombocytosis was lower than in patients without PNS, but without statistical significance. A shorter survival rate is consistent with previous reports [6,8,13].

Some limitations need to be acknowledged. Firstly, concerning the cohort size, although the total number of patients is substantial, the incidence of individual PNS is relatively low, resulting in a small number of patients divided by subcategories. Future studies in this field would benefit from focusing on multicenter studies to include a more significant number of patients. Secondly, this study is retrospective, and also we did not categorize patients based on their specific treatments for HCC. Thus, prospective studies are warranted to elucidate the dynamics of PNS appearance. These studies should use matching systems to assess the impact of PNS on survival within a larger cohort and follow how PNS influences survival depending on the treatment followed for HCC.

## 5. Conclusions

In conclusion, our study revealed a prevalence of PNS of 25.7% in HCC. The most common PNS observed was hypercholesterolemia. The presence of PNS in HCC strongly correlates with tumor size and AFP levels, with diabetes potentially serving as a risk factor. We have demonstrated that PNS occurrence is not uncommon in HCC and that paraneoplastic syndromes are associated with a poor prognosis and reduced survival. Specifically, paraneoplastic hypoglycemia tends to affect younger patients with larger tumor sizes and is associated with poorer survival. Erythrocytosis, hypercholesterolemia, and thrombocytosis also tend to be associated with shorter survival times, albeit without statistical significance, compared to patients without PNS or in matched patients. Therefore, in addition to assessing the usual prognostic factors, newly diagnosed patients with HCC should also undergo evaluation for the presence of PNS.

## Figures and Tables

**Table 1:** Demographic data of the subjects.

Parameters	PNS Positive *n* = 97	PNS Negative *n* = 281	*p* Value
Mean age (years)	64.18 ± 8.35	66.96 ± 8.83	0.0070
Gender (%)			
Females	17.5% (17)	33.5% (94)	0.0043
Males	82.5% (80)	66.5% (187)	0.0043
BMI (kg/m^2^)	25.5 (18.6–31.4)	22.5 (18.4–31.3)	0.6126
cACLD as the underlying disease (%)			
Yes	90.7% (88)	96.1% (270)	0.0741
No	9.3% (9)	3.9% (11)	0.0741
Child–Pugh grade (%)			
A [5–6]	44.3% (39/88)	35.6% (96/270)	0.1817
B [7–9]	23.9% (21/88)	39.3% (106/270)	0.0127
C [10–15]	31.8% (28/88)	25.1% (68/270)	0.2737
Etiology (%)			
ALD	22.7% (22)	18.1% (51)	0.4004
HBV	27.8% (27)	17.1% (48)	0.0331
HBV + HCV	0% (0)	2.2% (6)	0.3131
HBV + HDV	3.1% (3)	5.3% (15)	0.5469
HCV	33% (32)	46.6% (131)	0.0269
MASLD	13.4% (13)	10.7% (30)	0.5918
Number of HCC lesions			
1 lesion	41.2% (40)	52.7% (148)	0.0665
2 lesions	7.2% (7)	12.1% (34)	0.2506
3 lesions	0% (0)	1.1% (3)	0.7014
Multiple lesions	45.4% (44)	29.5% (83)	0.0063
Infiltrative (diffuse)	6.2% (6)	4.6% (13)	0.7242
HCC stage (BCLC) (%)			
A	12.4% (12)	14.9% (42)	0.6608
B	27.8% (27)	33.5% (94)	0.3622
C	30.9% (30)	27.4% (77)	0.5967
D	28.9% (28)	24.2% (68)	0.4345
PVT (%)	41.2% (40)	30.6% (86)	0.0743
Benign	2.5% (1/40)	10.5% (10/86)	0.2337
Malignant	97.5% (39/40)	89.5% (77/86)	0.2337
Patients’ comorbidities			
Arterial hypertension	44.3% (43)	37% (104)	0.2498
Other cardiac pathology	22.7% (22)	21% (59)	0.8350
Diabetes mellitus	37.2% (46)	29.2% (82)	0.1807
Chronic kidney disease	8.3% (8)	4.3% (12)	0.2112

Numerical variables with normal distribution are presented as means ± standard deviation, while variables with non-normal distribution are presented as median values and range; *n*—number; HCV—hepatitis C virus; HBV—hepatitis B virus; HDV—hepatitis D virus; ALD—alcohol-related liver disease; MASLD—metabolic dysfunction-associated steatotic liver disease; PVT—portal vein thrombosis; HCC- hepatocellular carcinoma; BCLC—The Barcelona Clinic Liver Cancer Staging System.

**Table 2:** Laboratory data of the subjects.

Laboratory Data				
Parameters	PNS Positive *n* = 97	PNS Negative *n* = 281	*p* Value	Normal Range Values
FBG (mg/dL)	109 (45–269)	116 (67–477)	0.0092	75–110 md/dL
Total cholesterol (mg/dL)	171 (45–523)	138 (19–216)	<0.0001	0–199 mg/dL
Platelet count (×10^3^/µL)	166 (36–971)	120 (37–392)	<0.0001	150–400 × 10^3^/µL
RBC (×10^6^/µL)	4.55 (2.6–7.2)	3.92 (1.6–5)	<0.0001	4–5.5 × 10^6^/µL
AFP levels (ng/mL)	218.7 (1.6–91,797)	45.5 (10–100,000)	0.0117	0–7.51 ng/ml

Numerical variables with normal distribution are presented as means ± standard deviation, while variables with non-normal distribution are presented as median values and range.; *n*—number; FBG—fasting blood glucose, RBC—red blood cells, AFP—alpha-fetoprotein.

**Table 3:** Child–Pugh distribution in paraneoplastic syndrome patients.

PNS	Child–Pugh A	Child–Pugh B	Child–Pugh C	*p* Value
PNS (+), *n* (%)	39 (44.3%)	21 (23.9%)	28 (31.8%)	*p* < 0.0001
PNS (−), *n* (%)	96 (35.6%)	106 (39.3%)	68 (25.1%)	
PNS (+) group				
Hypoglycemia	5 (12.8%)	5 (23.8%)	14 (50%)	
Hypercholesterolemia	20 (51.3%)	8 (38%)	10 (35.7%)	
Thrombocytosis	2 (5.1%)	4 (19.1%)	3 (10.7%)	
Erythrocytosis	12 (30.8%)	4 (19.1%)	1 (3.6%)	

Data are presented as a number and percentage; *n*—number; PNS—paraneoplastic syndrome.

**Table 4:** Etiology distribution in paraneoplastic syndromes patients.

PNS	ALD	HBV	HCV	Mixed Etiology	MASLD
PNS (+), n (%)	22 (22.7%)	27 (27.8%)	32 (33%)	3 (3.1%)	13 (13.4%)
PNS (−), n (%)	51 (18.1%)	48 (17.1%)	131 (46.6%)	21 (7.5%)	30 (10.7%)
PNS group					
Hypoglycemia	7 (31.8%)	4 (14.8%)	9 (28.1%)	2 (66.7%)	4 (30.8%)
Hypercholesterolemia	10 (45.5%)	14 (51.8%)	12 (37.5%)	1 (33.3)	4 (30.8%)
Thrombocytosis	3 (13.6%)	3 (11.2%)	4 (12.5%)	0	3 (23.1%)
Erythrocytosis	2 (9.1%)	6 (22.2%)	7 (21.9%)	0	2 (15.3%)

Data are presented as a number and percentage; PNS—paraneoplastic syndrome, ALD—alcoholic liver disease, HBV—B virus hepatitis, HCV—C virus hepatitis, MASLD—metabolic dysfunction-associated steatotic liver disease.

**Table 5:** Multivariate regression analysis results in patients with paraneoplastic syndromes.

Variables	OR	95% CI	*p* Value
Albuminlevels (g/dL)	4.21	1.56–18.92	0.035
PLT values (10^3^/µL)	1.09	1.00–1.22	0.086
Total bilirubin (mg/dL)	1.37	1.09–1.88	0.026
AFP levels (ng/mL)	1.71	1.11–2.23	<0.0001
Presence of diabetes mellitus	2.88	1.96–4.31	0.038
INR	1.11	0.89–1.98	0.541
Child–Pugh C	1.43	0.98–2.23	0.063
BCLC stage	0.94	0.79–1.22	0.231
MELD	0.88	0.81–1.12	0.32
Tumor size < 2 cm	0.91	0.87–1.33	0.0013
Tumor size < 5 cm	2.28	1.76–3.02	<0.0001

Data are presented as a number and percentage, or mean ± standard deviation; PLT—platelet count; AFP—alpha-fetoprotein; INR- international normalized ratio; BCLC—The Barcelona Clinic Liver Cancer Staging System; MELD—Model for End-Stage Liver Disease.

**Table 6:** Multivariate regression analysis results according to paraneoplastic syndrome’s type.

PNS+	Hypoglycemia			Erythrocytosis			Hypercholesterolemia			Thrombocytosis		
Variables	OR	95% CI	*p*	OR	95% CI	*p*	OR	95% CI	*p*	OR	95% CI	*p*
Albumin levels (g/dL)	3.61	1.96–16.42	0.0745	2.21	1.11–11.92	0.231	4.55	2.11–13.28	0.031	4.21	1.56–18.92	0.035
PLT values (10^3^/µL)	1.06	1.00–1.19	0.076	1.14	1.03–1.36	0.125	1.31	1.04–1.32	0.066	1.28	0.90–1.92	0.126
Total bilirubin (mg/dL)	2.31	1.92–4.88	0.263	1.07	1.01–1.83	0.021	1.57	1.33–2.88	0.082	1.70	0.98–2.13	0.034
AFP levels (ng/mL)	1.91	1.21–2.23	<0.0001	2.11	1.18–2.98	0.062	1.41	1.18–2.56	0.013	2.01	1.61–2.83	0.138
Presence of Diabetes mellitus	1.88	1.16–3.45	0.038	1.58	1.16–2.21	0.038	2.45	1.56–3.91	0.026	2.48	1.64–2.91	0.098
INR	1.41	0.89–1.68	0.671	1.01	0.59–1.34	0.741	1.13	0.59–1.48	0.0944	1.18	0.90–1.82	0.431
Child–Pugh C	1.23	1.08–2.43	0.163	1.52	1.11–2.36	0.083	1.43	1.28–1.88	0.098	1.53	1.13–1.94	0.103
BCLC stage	0.89	0.69–1.11	0.411	0.92	0.81–1.33	0.201	0.94	0.59–1.72	0.093	0.98	0.89–1.67	0.181
MELD	0.98	0.84–1.11	0.32	1.07	0.73–1.52	0.212	1.16	0.81–1.63	0.412	0.91	0.61–1.62	0.323
Tumor size < 5 cm	1.98	1.46–3.12	0.0531	2.18	1.91–3.43	0.0712	2.59	1.86–3.91	<0.0001	2.63	1.99–4.02	<0.0001

Data are presented as a number and percentage, or mean ± standard deviation; PLT—platelet count; AFP—alpha-fetoprotein; INR- international normalized ratio; BCLC—The Barcelona Clinic Liver Cancer Staging System; MELD—Model for End-Stage Liver Disease.

**Table 7:** Kaplan–Meier’s survival analysis.

Survival Time (Days)	Subjects without Paraneoplastic Syndromes *n* = 281		Subjects with Paraneoplastic Syndromes *n* = 97	
	Survival Proportion	Standard Error	Survival Proportion	Standard Error
30 days	0.794	0.0241	0.814	0.0395
60 days	0.719	0.0268	0.670	0.0477
90 days	0.654	0.2840	0.576	0.0530
183 days	0.539	0.0298	0.481	0.0510
365 days	0.436	0.0300	0.365	0.0454

## Data Availability

Data are available upon request.
